# Multiple Instances QoS Routing in RPL: Application to Smart Grids †

**DOI:** 10.3390/s18082472

**Published:** 2018-07-30

**Authors:** Jad Nassar, Matthieu Berthomé, Jérémy Dubrulle, Nicolas Gouvy, Nathalie Mitton, Bruno Quoitin

**Affiliations:** 1 HEI—Yncréa HdF, 59014 Lille, France; nicolas.gouvy@yncrea.fr; 2 Inria Lille—Nord Europe, 59650 Villeneuve d’Ascq, France; matthieu.berthome@inria.fr (M.B.); nathalie.mitton@inria.fr (N.M.); 3 Computer Science Department, University of Mons, 7000 Mons, Belgium; jeremy.dubrulle@umons.ac.be (J.D.); bruno.quoitin@umons.ac.be (B.Q.)

**Keywords:** Smart Grid, WSN, RPL, routing, QoS, objective function, metric

## Abstract

The Smart Grid (SG) aims to transform the current electric grid into a “smarter” network where the integration of renewable energy resources, energy efficiency and fault tolerance are the main benefits. This is done by interconnecting every energy source, storage point or central control point with connected devices, where heterogeneous SG applications and signalling messages will have different requirements in terms of reliability, latency and priority. Hence, data routing and prioritization are the main challenges in such networks. So far, RPL (Routing Protocol for Low-Power and Lossy networks) protocol is widely used on Smart Grids for distributing commands over the grid. RPL assures traffic differentiation at the network layer in wireless sensor networks through the logical subdivision of the network in multiple instances, each one relying on a specific Objective Function. However, RPL is not optimized for Smart Grids, as its main objective functions and their associated metric does not allow Quality of Service differentiation. To overcome this, we propose OFQS an objective function with a multi-objective metric that considers the delay and the remaining energy in the battery nodes alongside with the dynamic quality of the communication links. Our function automatically adapts to the number of instances (traffic classes) providing a Quality of Service differentiation based on the different Smart Grid applications requirements. We tested our approach on a real sensor testbed. The experimental results show that our proposal provides a lower packet delivery latency and a higher packet delivery ratio while extending the lifetime of the network compared to solutions in the literature.

## 1. Introduction

**Figure 1:**
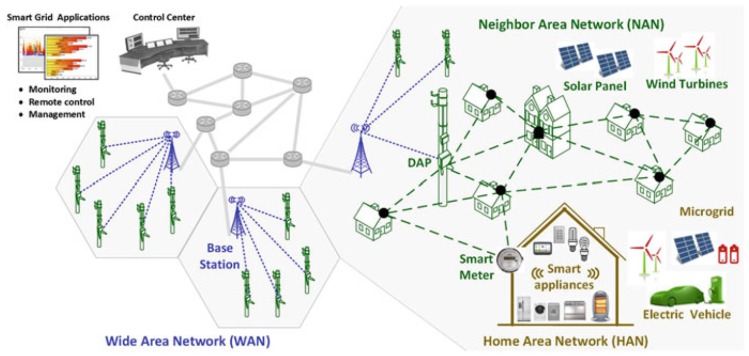
Smart Grid Communication Network [13].

Current electric grid no longer satisfies the need of energy of the twenty first century. The increased electricity offer per person is limited by the restrained electricity production and the aging and unsuitable infrastructures. This limitation is due to inaccurate management systems, inefficient operations and maintenance processes and a centralized communication system that lacks interoperability. Besides that, the introduction into the electricity grid of multiple sporadic Distributed Energy Resources (DERs) i.e., electric vehicles, photovoltaic cells, wind farms, located in sometimes unexpected places, makes the control of it even more complicated [1]. SG promises to solve these issues by operating with automatic control and operation in response to user needs and power availability improving efficiency, reliability and safety, with smooth integration of renewable and alternative energy sources. Managing the SG with a ubiquitous network to exchange regular and critical control messages all-over the power network becomes then crucial. Based on these observations and in order to shift from the existing electric grid to the SG, it appears necessary to instrument and master the high level and complex energy management on the electric grid. Consequently, one of the potential solutions envisioned is to equip the electrical grid with wireless sensors located at strategic measuring points to achieve remote monitoring, data collection and control of the grid [2]. Such sensors will constitute a parallel wireless data network to the electrical grid. A typical smart grid communication network consists of a Home Area Network (HAN), which is used to gather data from a variety of devices within the household, a Neighborhood Area Network (NAN) to connect smart meters to local access points, and a Wide Area Network (WAN) to connect the grid to the utility system as shown in Figure 1, the proposed WSN will operate mostly on HAN and NAN levels within this architecture.

**Figure 2:**
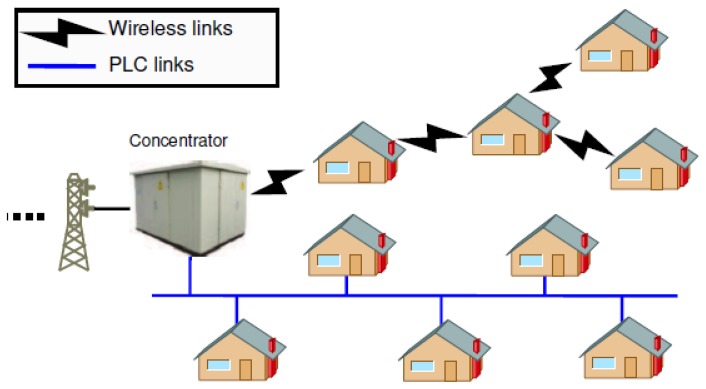
Smart Grid metering data collection.

SG applications are heterogeneous in terms of requirements, criticality and delay tolerance [3,4,5]. However, since these applications will generate different types of traffic (real-time, critical, regular) [6], they require different levels of QoS. Thus, for a wireless sensor network, different criteria have to be taken into consideration in order to achieve a proper communication with the following requirements: reliability, latency, auto-configuration, auto-adaptation, network scaling and data prioritization [6]. Among all the existing routing protocols used in the SGs, the IETF standard RPL [7] remains the most recognized and widely used [8,9]. As described in [10] RPL meets the scalability and reliability constraints of SG applications (e.g., Advanced Metering Infrastructure) and is recommended by the SG standards. Alongside with its support for wireless communications, RPL can be used with Power Line Communication (PLC) [11]. Figure 2 shows how smart meters (represented by houses) can send their measurements to the concentrator via wireless or PLC links. The same Media Access Control (MAC) layer can be compatible with a physical layer using wireless or PLC communications. We note that other protocols like LOADng [12] are used for SGs but this latter doesn’t support traffic differentiation which is an important aspect for SG applications.

As a general protocol, RPL is intended to meet the requirements of a wide range of Low-Power and Lossy Networks (LLNs) application domains including the SGs ones. It provides different QoS classes at the network layer through multiple logical subdivisions of the network called instances (more details in Section 2.1). RFC8036 [11] explains how RPL meets the requirements of SG applications and describes the different applications in SGs that can be done through RPL multiple instances. Following RPL, RFC8036 proposes five different priority classes for the traffic in SG AMI (Advanced Metering Infrastructure). Other papers classify the traffic into two levels: critical and periodic [14]. Based on that and since the traffic classes in the SG are not standardized, a single solution to route the traffic with different QoS may not be sufficient since the number of instances (traffic classes) vary depending on the application and the implementation. A multi-objective solution is thus essential to meet the QoS requirements of SG applications. Therefore, in this paper, we introduce OFQS an RPL-compliant objective function, with a multi-objective metric that considers the delay and the remaining energy in the battery nodes alongside with the quality of the links. Our function automatically adapts to the number of instances (traffic classes) providing a QoS differentiation based on the different Smart Grid applications requirements. We conducted real testbed experimentations which showed that OFQS provides a low packet delivery latency and a higher packet delivery ratio while extending the lifetime of the network compared to solutions in the literature.

The remaining of the paper is organized as follows: Section 2 presents first a brief overview of the RPL protocol. After that, prior works around the RPL protocol concerning the metrics and the multiple instances are provided. Finally, we present the motivations of using multiple instances in RPL. Section 3 describes our proposition in details. Section 4 shows the experiment setup and environment used to validate our proposition and its parameters. Section 5 presents the performance evaluation of our proposition and remaining issues are discussed in Section 6. Finally, Section 7 concludes the paper.

## 2. Related Work

### 2.1. RPL Protocol Overview

RPL is a Distance Vector routing protocol based on IPv6 for LLNs. It divides the network into multiple logical graphs called DODAGs (Direction-Oriented Directed Acyclic Graphs). DODAGs are tree-like structures oriented towards the root sink of the network built in order to avoid loops. Each node in a DODAG has a rank (hop-distance from the root), that increases by going down the tree from the root. RPL can use multiple overlapping DODAGs over the entire network to provide different levels of QoS in the network layer. In this case, each level/DODAG is called an instance. Thus an RPL network contains at least one instance. An instance is composed of one or more DODAGs. A node can join a single DODAG per example, but it can participate in multiple instances to carry different types of traffic simultaneously. An RPL instance is associated with an objective function in order to optimize the topology based on several metrics/constraints such as the shortest path or the quality of the links. Minimum Rank with Hysteresis Objective Function (MRHOF) [15] and Objective Function Zero (OF0) [16] are the two standardized objective functions in RPL. MRHOF uses the ETX metric [17] by default. OF0 uses the “step_of_rank” to compute the amount by which to increase the rank along a particular link using static (Hop count) or dynamic metrics (ETX). Whatever the metric, a DODAG construction starts from the root by sending DODAG Information Object (DIO) messages to its neighbors. The DIO contains the metric/constraint used by the objective function and the rules to join a DODAG (e.g., DIO sending interval). Nodes will receive and process DIO messages potentially from multiple nodes and make a decision to join the graph or not according to the objective function and local policies (if existing). Once a node joins a graph, it automatically has a route towards the sink through its parent node. The node then computes its rank within the graph, which indicates its position within the DODAG. If configured to act as a root, it starts advertising the graph information with the new information to its own neighboring nodes. If the node is a leaf node, it simply joins the graph and does not send any DIO message. The neighboring nodes will repeat this process and perform parent selection, route addition and graph information advertisement using DIO messages. At the end of this process, only upward routes (i.e., to the root) are built. To establish downward routes, a node must send a Destination Advertisement Object (DAO) to its parent containing prefix information of the nodes in its sub-DODAG, when the DAO message arrives to the root, the prefixes are aggregated and the downward routes are then built and made available to the parents, and so on. RPL nodes can also send DODAG Information Solicitation (DIS) messages to solicit DIO messages from neighbors. RPL uses the trickle algorithm to reduce the DIO messages rate. For example, if the number of DIO messages sent within an interval is not consistent with the network state, RPL resets the trickle timer to a minimum value. Otherwise, if the number of DIO messages is bigger than a certain threshold, the trickle interval (DIO message rate sending) is doubled up to a maximum value.

### 2.2. RPL Proposed Metrics and Modifications

Many researchers are active around RPL in order to adapt it to different Internet of Things applications. Moreover many critical analyses were made to highlight the gaps concerning reliability and adequate metrics in a SG environment [10,18,19]. ETX in MRHOF [15] and HC (Hop Count) in OF0 [16] are the two main metrics used in the objective functions. ETX finds paths with the fewest expected number of transmissions (including retransmissions) required to deliver a packet all the way to its destination [17]. Although ETX is reliable and widely used as a metric in wireless sensor networks, it does not take directly into account the latency which is critical in some SG applications [20]. ETX is not energy aware, thus for a link with few re-transmissions, ETX will keep sending packets on it without taking the decrease of battery nodes level into account. HC only takes the number of hops into consideration to calculate the best path which is not always satisfactory in LLN.

In [21] several routing metrics were proposed to be used for path calculation in LLN, i.e., the Throughput, Node Energy, Latency, Link reliability with the LQL (Link Quality Level) or ETX metric. An energy-based objective function for RPL that uses the remaining energy as the main routing metric was proposed in [22]. It achieves a better load balancing compared to ETX and increases the network lifetime but with a lower delivery ratio. In [23], the authors proposed NL-OF, an objective function based on a non linear length that construct DODAGs from roots to nodes such that the non linear length is the smallest possible. They evaluated it using Cooja while considering three QoS parameters: End-to-end delay, packet loss and jitter. In [24] two MAC aware routing metrics were proposed to be used in RPL: R-metric and Q-metric. R-metric extends ETX by considering packet losses due to the MAC contention. Q-metric provides load balancing by selecting the lightest parent in terms of traffic load by solving an optimization problem and mainly considering reliability, transmission and reception power consumption. ETT-LB was proposed in [25]. It is based on the ETT (Expected Transmission Time) metric [26], which extends ETX by considering the link transmission rate and packet size, adding to it the Expected Delay Time (EDT), which is the average link load at a node in order to achieve load balancing. In [27] $L^{2}AM$ metric was proposed. It is based on an combination of both data reliability (defined by ETX) and the nodes residual energy. Although their solution extended the network lifetime, it remains not adapted to a network with heterogeneous applications in terms of criticality and powered/battery nodes. Fuzzy logic metric combination was also considered in several works [28,29,30] in order to be used for RPL. They combined several metrics like end-to-end delay, HC, link quality and battery level. In [31] two combinations of two metrics were proposed: lexical and additive. In the lexical combination, the second metric is inspected only if the first one leads to equal paths, while in the additive combination the paths are calculated based on a different cost given to each metric. Multiple instances in RPL and QoS were studied in many works [9,32,33]. Yet, these works limit the number of instances to two and don’t take into consideration the drawbacks of the used metrics (ETX and HC) concerning the energy efficiency and end-to-end delay.

As a conclusion, a single routing metric cannot assure traffic differentiation in a SG since different applications require different QoS levels. In addition, in a multiple instance environment, the chosen objective function/metric has to guarantee the QoS requirements of the concerned SG application, which to the best of our knowledge has not been proposed yet. This is why we propose OFQS with its multi-objective metric mOFQS taking account of these requirements and improving the communication in the SG. Finally, note that OFQS, by integrating the different requirements of the SG applications, is suitable for any other application with these same demands and criticality variations e.g., Smart City applications.

### 2.3. Why Multiple Instances?

SG applications are heterogeneous in terms of requirements, criticality and delay tolerance [3,4,5,34]. Guaranteeing that each of these applications meets its QoS demands requires a multi-objective solution. As an example, we can cite some of the following main SG applications and their requirements.

- Advanced Metering infrastructure (AMI) consists of an integrated system of smart meters for measuring, collecting, analyzing and communicating energy consumption of smart appliances. Enabling two-way communication between utilities and customers and providing a number of important functions that were not previously possible or had to be performed manually, such as the ability to automatically and remotely measure electricity use, connect and disconnect to a service, identify and isolate outages, and monitor voltage.
- Demand Side Management (DSM) consists of a set of interconnected and flexible programs which grants customers a greater role in shifting their own demand for electricity during peak periods, and reducing their overall energy consumption. DSM comprises two principal activities:
  - Demand Response (DR) or load shifting which aims to transfer customer load during periods of high demand to off-peak periods. The grid operator or other stakeholders influence the customers behavior mostly by monetary incentives, allowing them to participate in the energy market competition by changing their energy consumption approach instead of being passively exposed to fixed prices, which results in profits for both, the companies and the end-users.
  - Energy efficiency and conservation programs which allow customers to save energy while receiving the same level of end service, such as when they replace an old electric appliance with a more energy efficient model.
- Distribution Automation (DA) is defined as the ability of taking an automated decision to make fault detection, more efficient isolation and restoration in a grid by remotely monitoring, controlling, manipulating and coordinating distribution, improving then the reliability accross the grid. DA offers new functionalities, incorporate alarming and automated feeder switching, which in turn will help reduce the frequency and duration of customer outages. Substation automation is achieved through Supervisory Control and Data Acquisition (SCADA) systems which are able to make these automated decisions in real time by running algorithms based on the data they receive and orchestrate adjustments to optimize voltages and self-heal any failure issues.
- Distributed Energy Resources (DERs) such as photo voltaic cells, wind turbines and energy storage points present one of the main benefits in a SG. These DERs will be able to supply particular areas with electricity when they are isolated from the main power grid due to failure conditions or system and equipment failures. Moreover, these DERs foster the shift from a centralized power system towards a more decentralized system by contributing to the evolution of local grid areas served by one or more distribution substations and supported by high penetrations of DERs called microgrids.
- Electric transport via electric vehicles (PEV: Plug-in Electric Vehicles) or hybrid electric vehicles (PHEV: Plug-in Hybrid Electric Vehicles) aims to improve or even replace traditional transport by reducing emissions produced by fossil fuels. For that, an electric vehicle uses one or more electric motors that are powered by a rechargeable electric accumulator. SGs can better manage vehicle charging so that rather than increasing peak loads, the charging can be carried out more strategically, when for example electricity demand is low or when the production of renewable electricity is high. In the long run, SGs can use electric vehicles as batteries to store renewable and other sources of electricity for later use.

However, since these applications will generate different types of traffic (real-time, critical, regular) [6], they require different levels of QoS. Table 1 shows the diversity of the delay tolerance and reliability for the different NAN applications [5]. Thus, for a wireless sensor network, different criteria have to be taken into consideration in order to achieve a proper communication with the following requirements: reliability, latency, auto-configuration, auto-adaptation, network scaling and data prioritization [6]. From here the need of an objective function with multi-objective metric for RPL.

## 3. Proposed Solution

### 3.1. OFQS Objective Function

To overcome the lacks of the metrics traditionally used by RPL and allows the multi-instances, we introduce the tunable multi-objective metric mOFQS to be used by OFQS. The mOFQS metric adapts automatically to the number of instances in the network depending on their criticality level by tuning its parameters jointly. OFQS is derived from MRHOF as it relies on the same rank calculation mechanism, it adopts hysteresis to prevent routing instabilities by reducing parent switches under a certain threshold.

### 3.2. QoS Factors in OFQS

OFQS with its metric mOFQS takes the quality of the links into consideration by calculating their ETX value. In Contiki Operating System, ETX is implemented in the MRHOF objective function. ETX is updated based on callbacks from the MAC layer which gives the information whether a MAC layer transmission succeeded, and how many attempts were required. Lower ETX values mean better links quality to route the packets with less re-transmissions. Alongside with the quality of links, the delay is an important factor in SG applications as already mentioned. For that, mOFQS considers the delay *d* between sending the packet and receiving it in the network layer between two adjacent nodes. This allows the algorithm to choose faster links especially for critical applications considering at once transmission, queuing and interference delays. Moreover, in a SG, electricity and energy do exist, but connecting sensors to such high voltage with intermittent and ill-adapted energy levels is sometimes inappropriate or physically impossible. For that, battery-powered sensors must be deployed all over the grid alongside with the mains powered ones. Different requirements for different applications may tolerate in some cases passing by a longer route in order to preserve the remaining energy in the nodes. Hence, considering the battery level for the nodes in our metric will be beneficial in terms of traffic load balancing and network lifetime. To do so, we classify the remaining energy in the nodes into three Power States (PS) [35]:

- PS = 3: Full battery state (ranging between 100% and 80%) or main powered
- PS = 2: Normal battery state (ranging between 80% and 30%)
- PS = 1: Critical battery state (less then 30%)

By using this classification, weak nodes become unfavorable in the route selection by penalizing the ones with a smaller PS. We note that these thresholds could be adjusted for other applications depending on the network characteristics.

### 3.3. *mOFQS* Metric

**Figure 3:**
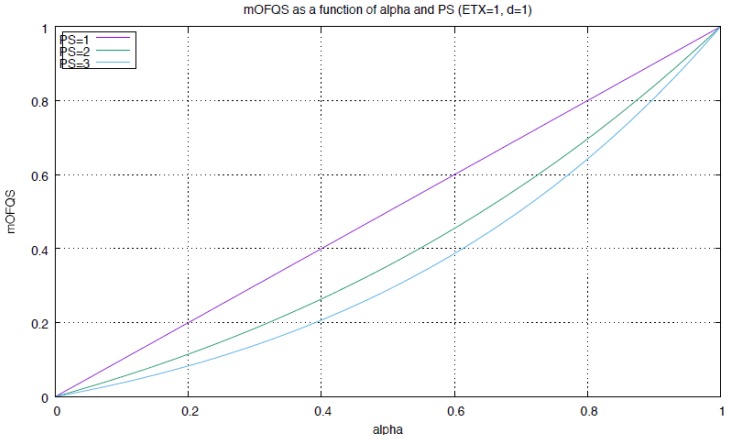
mOFQS variation with α.

To enable RPL to consider the remaining energy, the latency and the multiple instances beside the reliability using ETX, mOFQS includes the Power State PS, the delay *d* of delivering a packet within two nodes in milliseconds and two parameters α and β. mOFQS formula is shown below:

$$\mathrm{mOFQS}=\frac{\alpha (\mathrm{ETX}\times d)}{PS^{\beta }}$$

where α and β are two tunable parameters with α=1−β, 0<α<1 and 0<β<1. mOFQS is an additive metric whose values over the path is the sum of the values at each hop. The idea is to multiply ETX by the delay *d* for every hop to get the links reliability while considering the delay of the packet delivery, then multiply the factor ETX×d by α to foster link quality and end-to-end delay for critical applications by increasing α. α(ETX×d) is then divided by PS to the power of β. Increasing or decreasing β will similarly foster PS. If the application is critical, β should be decreased (resp. α increased). For delay tolerant applications, increasing β will result in a longer route while conserving the nodes power since the metric will weight more node energy level rather than link quality or end-to-end delay. Figure 3 shows how mOFQS behaves as a function of α for the different PS values (with ETX = 1 and *d* = 1). The higher α values and the more critical energy level (the worst the conditions), the higher the mOFQS value to be considered.

**Figure 4:**
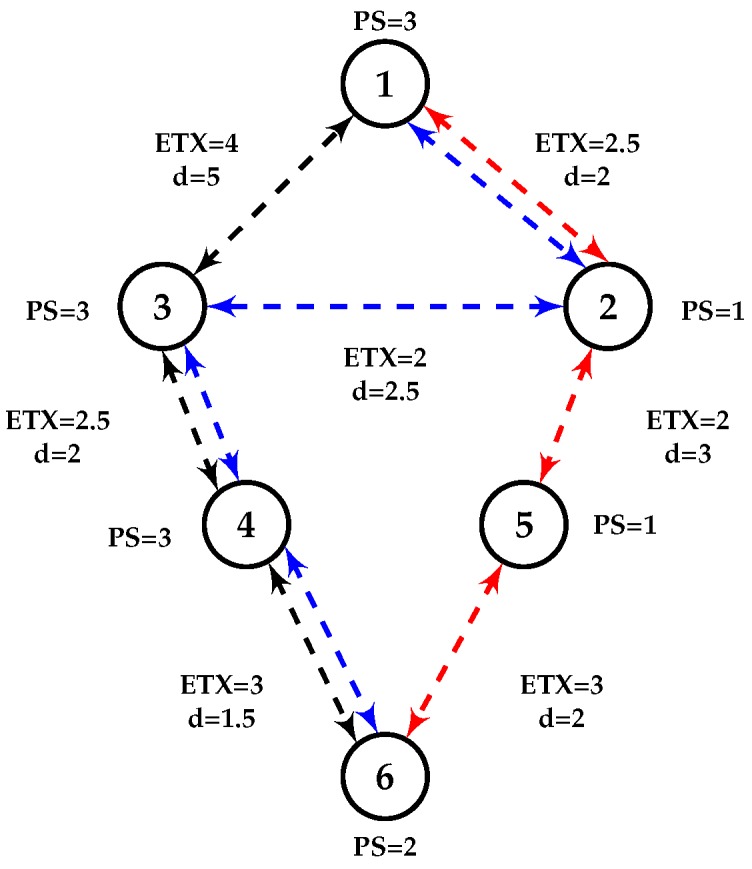
Network with different ETX, delay *d* (in ms) and PS values.

Each node chooses the path upward in its DODAG with the lowest value provided by mOFQS. As mentioned, the lowest value of mOFQS defines the best quality links. First of all, varying α and β allow us to differentiate between instances depending on their criticality level. Less critical applications will tolerate the use of less good links. Dividing α(ETX×d) by ${\mathrm{PS}}^{\beta }$ aims to foster routes where the nodes consumed less their batteries or are main powered. For one application, we favor α or β against the other, and since α+β=1, when one parameter increases the other decreases and vice-versa. Figure 4 depicts a small network of 6 nodes running RPL, considering two different applications: one is critical and belongs to Instance 1 and the other is regular and belongs to Instance 2. When node 6 needs to send a packet to node 1, we consider the following paths: path 1: 6→5→2→1 or path 2: 6→4→3→1 or path 3: 6→4→3→2→1. Table 2 shows the different paths metric values with ETX, HC and mOFQS. For ETX alone, path 1 is the optimal one since it is the only metric used. We can thus note that each path features different QoS and can be favored by using a metric rather than another one. This is how we will achieve the multi-instance routing and QoS differentiation. For ETX & HC, ETX is used for the critical traffic (Instance 1) and HC for the regular one (Instance 2), as we can see Instance 2 optimal path will be 1 or 2 since they count less hops, and for Instance 1, it will be path 1 which has ETX = 7.5. Neither ETX or HC take energy consumption and delay into consideration, unlike mOFQS where α and β values will foster one path over the other. With mOFQS, in Instance 1 with critical traffic which requires minimal latency, we have to route the packets as fast as possible while guarantying a reliable link. Thus, we increment α (α = 0.9) fostering ETX×d (reliability and latency), which means decreasing β (β = 0.1). mOFQS fosters path 1 since it has better ETX and *d* values than paths 2 and 3. In Instance 2, where the traffic is not critical, we increment β (β = 0.9) and foster PS, which means that we might pass by a longer and less reliable route, while guaranteeing load balancing. Consequently forcing paths where nodes consumed less their batteries (path 3 where node 3 and 4 have more than 80% energy left in their batteries unlike path 1 where nodes 2 and 5 have less than 30% energy left). We achieve then a traffic distribution along the nodes by passing by path 3 and extending the network’s lifetime.

### 3.4. Instances Classification

Traffic classes in SG are not yet standardized. In this paper, we use the classification presented in [5] for the requirements in terms of delay and reliability in a Neighborhood Area Network (NAN) as shown on Table 1. The aforementioned classification sorts the traffic into 9 different classes, ranging from delays inferior than 3 s with reliability >99.5% for the most critical class to delays of hours/days with a reliability of >98% for the least critical class. In our model, we have gathered these 9 classes into 3 classes with 3 main instances:

- Instance 1: critical traffic with an authorized delay ranging between 1 and 30 s and a reliability of >99.5% packets received with α = 0.9 and β = 0.1
- Instance 2: non-critical traffic with an authorized delay of days and a reliability of >98% packets received with α = 0.1 and β = 0.9
- Instance 3: periodic traffic with an authorized delay ranging between 5 min and 4 h and a reliability of >98% packets received with α = 0.3 and β = 0.7

In this classification, we increment α for the critical traffic thus fostering the link quality and end to end delay assured by ETX and *d*, which results in routing the packets in a reliable and faster path. For less critical traffic we increment β which leads to fostering paths where the nodes consumed less their batteries and then achieving a load balancing. We note that our model is not limited to this classification and for any other one α and β can be modified or be totally independent depending on the network characteristics.

## 4. Experiment Setup

In this section, we detail our network setup and provide a quick overview about the wireless sensor testbed used to validate our proposition.

### 4.1. FIT IoT-LAB Testbed

**Figure 5:**
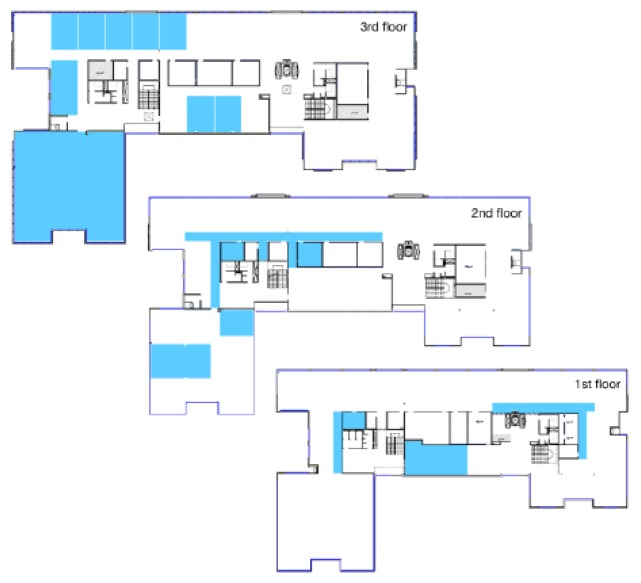
Topology of the deployment on FIT IoT-LAB Lille’s site (https://www.iot-lab.info/lilles-new-physical-topology-released/).

FIT IoT-LAB [36,37] provides a large scale infrastructure facility and experimental platform suitable for testing small wireless sensor devices and heterogeneous communicating objects. It provides full control of network nodes and direct access to the gateways to which nodes are connected, allowing researchers to monitor several network-related metrics. FIT IoT-LAB features over 2000 wireless sensor nodes spread across six different sites in France. For our experimentation, we chose nodes from the site of Lille. These nodes are distributed inside a 200 m${}^{2}$ room and on the different corridors of the Inria building, enabling a large-scale multi-hop topology (Figure 5).

### 4.2. Battery Level Measurement

**Figure 6:**
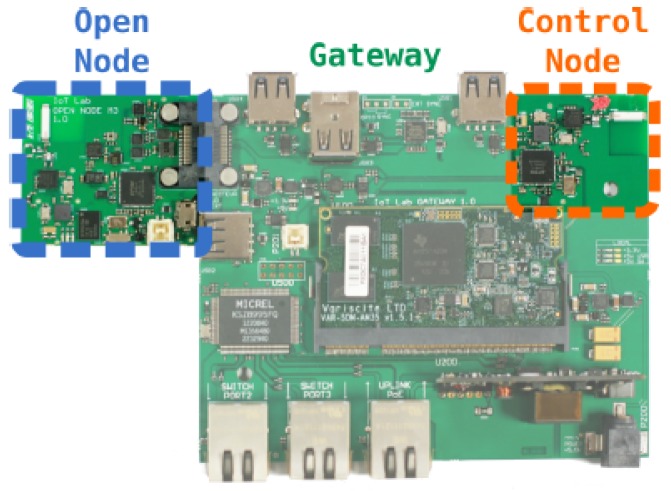
Hardware of an IoT-LAB node [36].

Each node from the FIT IoT-LAB platform is composed of three parts as shown in Figure 6:

- the gateway that is responsible for flashing the open node and connecting it to the testbed’s infrastructure
- the open node that runs the experiment firmware
- the control node that runs radio sniffing and consumption measurement

Because we needed to run scenarios with varying battery levels on different nodes, it was impractical to rely on actual lithium batteries. Instead, we relied on the real-time consumption measurement performed by the control node. The gateway collects consumption measurements every 140 μs, and write Orbit Measurement Framework (OML) files, with a μs time stamped value of the power consumption of the open node in Watts.

A software running inside the testbed’s user area was then collecting these consumption files for each node in the experiments, and numerically integrating the values through a basic rectangle sum. At the beginning of each experiment, the battery capacity of each node was decided randomly between two different values. During the experiment, when a node’s consumed virtual battery exceeded the virtual battery capacity, the node was electrically shutdown by the gateway. The network must then reorganize without the missing peer. The experiment was stopped when at least 20% of the nodes ran out of battery. The integrated total consumed energy in Joules, as well as the battery percentage, were sent to each node through its serial port using the gateway’s tooling that replicates the open node serial port on an accessible TCP socket. A Contiki process received this information on the node, which is used afterwards in the metric computation and route calculation. For real-life application of this paper in an actual sensor network, devices would be fitted with an adequate interface to their battery controller subsystem, which would be queried by the Contiki’s application through an I2C, SPI or similar link. We note that the physical environment conditions that may influence the discharge and lifetime of the batteries [38,39] are out of scope of this paper.

### 4.3. Network Setup

To evaluate our approach on FIT IoT-LAB, the experiment was performed on Contiki OS using M3 nodes. The topology consists of 67 client nodes that send UDP packets to the server repeatedly on an interval of 1 to 60 s between two subsequent transmissions in order to differentiate the sending rate between the two instances. Experimentation parameters are presented in Table 3. Multiple RPL instances are not fully supported in Contiki, we used an implementation (https://github.com/jeremydub/contiki) [40] where multiple instances are supported. We implemented it on FIT-IoT lab in order to evaluate our proposition. In this new RPL implementation, nodes can participate in multiple instances with different objective functions and metrics. A specific instance can be set at application layer, allowing traffic differentiation. It also supports new constraints in DIO metric container object. Also, a root can now be a sink for multiple applications that have different route requirements. For our experiments, we considered the upward traffic with two instances: OFQS with critical and periodic traffic (Instance 1 and Instance 3 resp.) as presented in Section 3.4 compared to RPL with MRHOF/ETX for critical traffic and OF0/HC for periodic traffic. All experiments results are measured within a 90% of confidence interval.

## 5. Performance Evaluation

In this section, we evaluate our proposition OFQS in comparison with MRHOF/OF0 in terms of four performance metrics: End-to-end delay, network lifetime, load balancing and packet delivery ratio. It is important to mention that our approach is not specific to SGs but it is mostly suitable to any context with different applications on the same physical topology with different characteristics/QoS. SGs are only an example of such applications. We note that in addition to the preliminary results obtained by simulation and available at [41], these experimentation results provide a large scale evaluation of our metric in real environment.

### 5.1. End-to-End Delay

**Figure 7:**
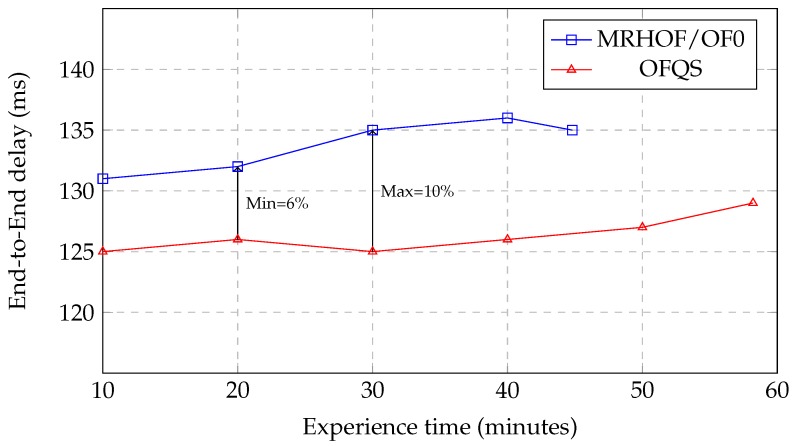
End-to-End delay variation with time.

Delay is considered when selecting the best next hop according to mOFQS. To evaluate the End-to-End delay, we calculated the difference in time between sending a packet by the client and the reception by the server. We actually ran several tests in order to check the synchronization of the clock, and we realized that clock drift is negligible. Figure 7 shows the end-to-end variation throughout the experience time for both MRHOF/OF0 and OFQS. We can see that OFQS end-to-end delay is always below MRHOF/OF0 with an improvement ranging from 6% to 10%. Even though HC chooses paths with the fewer hops from the sink, these paths are generally slower with a higher potential of loss since HC is not aware of links congestion and saturation. On the other hand, ETX is not also aware of the delays due to interference on the links and queuing in the nodes as long as the packets are transmitted; therefore, sending a packet with less re-transmissions does not necessarily mean sending it on a faster link. In OFQS, the *d* factor takes into account the delay of sending a packet between two adjacent nodes in the metric computation. In this way and mainly in instance 1, the metric will foster faster routes with less interference and congestion that HC and ETX are not aware of. Moreover, we can see that the delay variations for OFQS are minimal between 20 and 40 min. This is due to the variation of the battery levels (PS passing to a smaller value) which affects the choice of routes with low delays. Finally, and starting from the 40th min until the end of the experiment, we can notice that the end-to-end delay starts to increase. This is due to the depletion of the batteries of some nodes that switch to a lower PS, which means that the metric will switch from these nodes to other ones and foster sometimes longer routes in order to increase the network lifetime. We note that the experience stops after 44 min for MRHOF/OF0 compared to 58 min for OFQS as we can see on the graph. This extension of the network lifetime will be discussed in detail in Section 5.2.

### 5.2. Network Lifetime and Load Balancing

**Figure 8:**
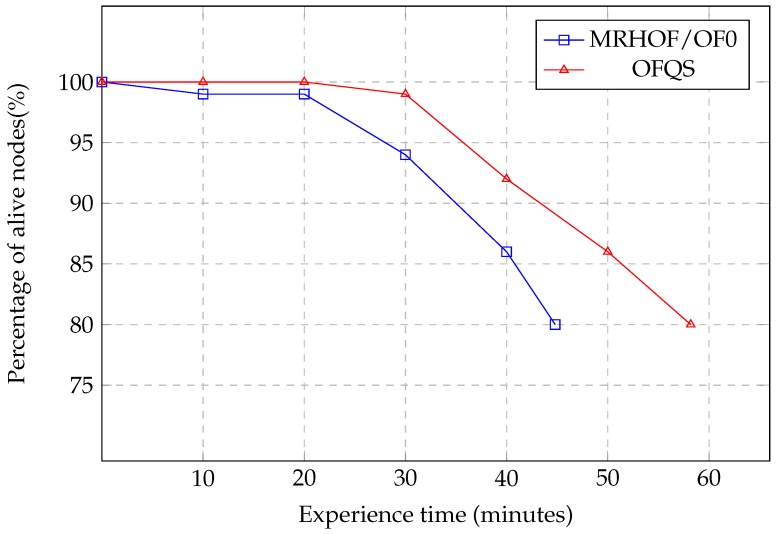
Network lifetime variation.

**Figure 9:**
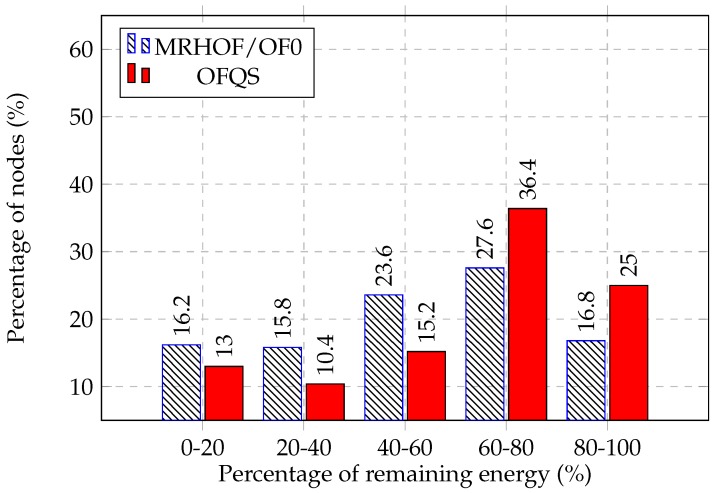
Remaining energy distribution among the nodes after 30 min.

Figure 8 shows the percentage of alive nodes for both MRHOF/OF0 and OFQS within the experience time. We observe that for MRHOF/OF0 and after 10 min, battery nodes started to drain reaching the threshold of 20% after 44 min. Concerning OFQS and for the first 20 min, all the nodes are still functional and none has consumed its total battery. After that time, the batteries started to drain reaching 20% of dead nodes after 58 min. OFQS achieves a gain of 14 min of network lifetime increase which is around 25% more than the one achieved by MRHOF/OF0. This gain is due to the power state that is taken into consideration in OFQS. In the same way, we can see in Figure 9 that after 30 min of the experiment, 16.2% of the nodes have a battery level between 0 and 20% in MRHOF/OF0 compared to 13% for OFQS. While 61.4% of the nodes in OFQS have a a battery level between 60% and 100% compared to 44.4% in MRHOF/OF0. This shows that in OFQS, PS is switching to nodes that consumed less their batteries achieving then a better load balancing of traffic among the nodes. In fact, mOFQS does not take into consideration the rate of battery depletion from the beginning. In the initial state, where all batteries are fully charged, the metric will pick paths without battery level consideration since they are all fully charged. During the experience, the most loaded nodes will undergo a quicker battery drain than others and thus the power state changing (PS = 3-> PS = 2). Here mOFQS will react and switch to other nodes that consumed less their batteries achieving thus an extension of the network lifetime and a better load balancing.

### 5.3. Packet Delivery Ratio

OFQS achieves 91.8% of Packet Delivery Ratio (PDR) compared to 85.7% for MRHOF/OF0. This shows that OFQS overpasses MRHOF/OF0 in terms of reliability. Firstly, HC has no link reliability mechanisms in the route selection which causes packet loss by selecting congested paths. Moreover, although ETX considers the link reliability, mOFQS still overpasses it by considering the delay of sending a packet in one hop which reflects the interference and the queuing delay on that hop by multiplying ETX×d, allowing then more reliable routes to be chosen.

## 6. Discussion

Before coming to our conclusions, we discuss some relevant issues in our proposition. While OFQS proved its efficiency in the experiments, a few things still need to be further investigated. In our instances classification (Section 3.4), the parameters α and β were fixed for the three instances. This selection could be optimized and made dynamic using machine learning or fuzzy logic techniques in order to compute the most suitable classification for every traffic class. These techniques should respect the constraints of the Wireless Sensor Network in terms of energy and computational limitations. Furthermore, the multiple instances in RPL aim to differentiate the traffic in the network. Further analysis should be made in order to study the impact of one instance on another while running together on the same network, and how many instances can we maximum run by still ensuring a proper traffic differentiation between the instances.

## 7. Conclusions

In this paper, we have proposed a new objective function to be compliant with RPL to support the multi-instance approach proposed by the standard. Our approach takes into consideration different features of both nodes and links and is compliant with the standard. We have run the experiment using realistic settings and results show the high performances of OFQS It achieves significant improvement in terms of End-to-End delay, network lifetime and PDR while insuring a load balancing among the nodes compared to standard solutions. In the future, we intend to investigate open issues discussed in Section 6.

## Figures and Tables

**Table 1:** NAN requirements in terms of reliability [5].

Data Traffic	Maximum Allowed Delay	Reliability
DA-Data related to the protection of the distribution network	<3 s	>99.5%
DERs (Distributed Energy Resources)—Data related to the protection of the distribution network	<4 s	<99.5 %
Critical traffic of: DA, DSM, AMI, DERs	<5 s	>99.5%
Electric transport	<10 s	>98%
Non critical traffic of DSM & AMI	<15 s	>98%
Non critical traffic of DA & AMI	<30 s	>98%
Network configuration traffic, normal AMI traffic	<5 min	>98%
Normal AMI traffic	<4 h	>98%
Network configuration traffic	<Hours/Days	>98%

**Table 2:** Paths values for the different metrics used.

	Paths		
	Path 1	Path 2	Path 3
**Metrics**	6->5->2->1	6->4->3->1	6->4->3->2->1
Instance 1	7.5	9.5	10
ETX			
Instance 2	-	-	-
Instance 1	7.5	9.5	10
ETX			
Instance 2	3	3	4
HC			
Instance 1	14.9	23.9	16.3
mOFQS			
α = 0.9 β = 0.1			
Instance 2	1.4	1.2	1.1
mOFQS			
α = 0.1 β = 0.9			

**Table 3:** Parameters of the experimentation.

Parameters	Values
OS	Contiki master version
Testbed	FIT IOT-LAB
Communication protocols	CSMA, RDC contikimac, IEEE 802.15.4, ContikiRPL, IPv6
OF	1-OFQS with 2 instances
	2-MRHOF (ETX) & OF0 (HC)
Number of nodes	67 clients and 1 server
Sensors	M3
Microcontroller Unit	ARM Cortex M3, 32-bits, 72 MHz, 64 kB RAM
Maximum packet size	30 kb
Sending interval	1 packet every 1 to 60 s
